# Preserving Patient Stories: Bioethical and Legal Implications Related to the Shift from Traditional to Digital Anamnesis

**DOI:** 10.3390/clinpract14040095

**Published:** 2024-06-21

**Authors:** Filippo Gibelli, Paolo Bailo, Giuliano Pesel, Giovanna Ricci

**Affiliations:** Section of Legal Medicine, School of Law, University of Camerino, 62032 Camerino, Italy; filippo.gibelli@unicam.it (F.G.); dr.giuliano.pesel@gmail.com (G.P.); giovanna.ricci@unicam.it (G.R.)

**Keywords:** anamnesis, digitalisation, bioethics, medicolegal, patient narratives, healthcare technology, electronic health records, medical ethics, information privacy, health informatics

## Abstract

It is since the beginning of the so-called ‘digital revolution’ in the 1950s that technological tools have been developed to simplify and optimise traditional, time-consuming, and laborious anamnestic collection for many physicians. In recent years, more and more sophisticated ‘automated’ anamnestic collection systems have been developed, to the extent that they can actually enter daily clinical practice. This article not only provides a historical overview of the evolution of such tools, but also explores the ethical and medico-legal implications of the transition from traditional to digital anamnesis, including the protection of data confidentiality, the preservation of the communicative effectiveness of the doctor–patient dialogue and the safety of care in patients with poor digital and health literacy.

## 1. Introduction

In the context of medical practice, the anamnesis (also called medical history) represents an absolutely fundamental and indispensable moment, as it is the tool that allows the caregivers to form a complete and exhaustive picture of a patient’s health condition. In the absence of a correct and accurate anamnesis, it is basically impossible to implement an appropriate planning of the diagnostic and therapeutic pathway. In fact, it is well known that defects in the collection of the anamnesis are often at the origin of adverse events [1,2,3]. It is estimated that the collection of a correct and complete anamnesis, carried out using appropriate communication techniques, enables a correct diagnosis to be made in 76% of cases [4].

Traditional anamnesis collection is carried out through an interview between the physician and the patient, but over the past 70 years, with the advent of the digital age, more and more innovative modes of anamnestic collection, based on technological tools, are emerging. So-called “digital medical history” or “digital anamnesis” harnesses technology to collect, store, and analyse patient data, including by examining the documentation represented by electronic medical records and the outputs of wearable devices. 

The digital anamnesis looms as a potential tool to ensure greater completeness of anamnestic collection and a reduced workload for healthcare professionals, while at the same time weakening the doctor–patient interrelationship, since the patient can fill out the anamnestic questionnaire in total autonomy, even from home, using digital tools. It is evident that the transition from traditional to digital anamnesis raises important medico-legal and bioethical issues. This article aims to explore these implications by comparing and contrasting digital anamnesis with its classical counterpart, in order to shed light on the challenges and opportunities presented by this digital evolution of healthcare.

## 2. The Traditional Anamnesis: Rationale and Critical Aspects

The term anamnesis derives from the ancient Greek “anamnesis” (a calling to mind) and consists of the collection of information about the patient’s current and past health status. In antiquity, the first work on anamnesis that has come down to our times was by Rufus Ephesius (150 BC–80 BC). This work was published and translated from Greek into French by Daremberg and Ruelle in the 19th century, and the term anamnesis only came into use in the mid-19th century in German-speaking countries and the Netherlands [5]. Traditionally, the anamnesis takes place orally, and is developed through a series of questions asked by the physician to the patient (or to family members, friends, or witnesses of a particular event in the case of special situations, such as in the case of mentally unreceptive individuals, or victims of an event they do not remember, or unconscious patients).

In order to understand the centrality of medical history collection in the diagnostic-therapeutic process, it is appropriate to quote a phrase conventionally attributed to William Osler (1849–1919), a famed Canadian physician and one of the fathers of modern medicine: “Listen to the patient. He is telling you the diagnosis” [6]. Generally, the anamnesis is divided into five parts:

- Family history: The current and past state of health of surviving relatives is investigated, as well as the causes of death of deceased relatives;
- Personal history: Its aim is to find out the patient’s general characteristics and lifestyle habits, namely weight and height, dietary regime, alcohol and drug consumption, smoking habits, sporting activity, sexual habits, drugs and supplements taken, any allergies or intolerances to drugs, foods or environmental substances, regularity of bowel and urinary function, current and previous employment, childbirth patterns, psychomotor development in childhood, compulsory military service (for males), any pregnancies (for females);
- Past history: The patient’s entire medical history is investigated, from birth to the time of the anamnesis. In particular, all illnesses, surgeries, and traumas suffered by the patient must be investigated;
- History of presenting symptoms: The reason why the patient came to the attention of the doctor is investigated specifically and in a high degree of detail. It is important at this stage to gather information about any similar incidents in the past and to have the patient explain how he or she managed the onset of symptoms before seeking medical attention, and with what results;
- Current status: The symptoms and signs presented by the patient at the time of the anamnestic investigation are thoroughly investigated.

The collection of the medical history is as fundamental as it is difficult to do optimally. This is primarily because it is a time-consuming activity. There is no codified time cut-off indicating per se whether and how adequate the history collection is, varying greatly from case to case. The anamnesis of a young, healthy subject without pathologies and previous traumas or interventions may be absolutely complete even if collected in a few minutes. On the contrary, the history of an elderly person, perhaps with difficulty in expressing himself/herself and slow speech, and with a rich past medical history may take more than 30 min. For example, in a 2017 paper, a Turkish group conducted a study to determine the minimum appropriate duration for the examination of patients in the pulmonology setting. The investigation revealed that, of all examination phases, the collection of the medical history took the longest time, with an average duration of 5.0 ± 3.6 min (minimum: 0.25, maximum: 36.6 min) [7].

The questions that should be asked are: does the doctor have sufficient time to conduct adequate anamnestic investigations? And above all, does he/she have the appropriate communication skills to be able to carry out a high-quality anamnestic collection?

As early as 1972, Slack and Slack [8] wrote about traditional anamnesis: “Dialogue between doctor and patient is a time-honored process revered by the medical profession. During conversation with his patient the doctor can establish rapport, evaluate his patient’s ability to engage in productive discussion, observe his patient’s nonverbal behavior and collect historical information of clinical relevance … doctors as interviewers are busy, expensive and sometimes hard to find. It seems reasonable, therefore, to look for substitutes that will serve at least some of the purposes of medical interviewing in widespread and inexpensive ways” [9]. 

What emerges from the analysis of Slack and Slack, true pioneers in interactive history taking, is that doctors tend to be bad interviewers, because they have neither the time nor the communication skills to conduct an optimal interview.

The communication aspect is also paramount. The possession of good communication skills on the part of the physician has been shown to predict a higher level of patient satisfaction, resulting in a higher likelihood of adherence to the treatment programme [10,11]. The moment of the collection of the medical history represents the first real contact between doctor and patient, i.e., a moment in which the two actors in the therapeutic relationship interface by establishing a connection, including an emotional one, between them. Underlying a quality anamnestic collection is a whole series of precise communication rules, which are far from obvious, and for which preparation and a considerable share of individual sensitivity are required. These rules are effectively illustrated by Priscilla Peart [12]: speaking clearly, ensuring that the patient can hear without the need to shout, unless they have a significant hearing impairment, using open-ended questions initially to collect comprehensive information, then transitioning to closed questions to refine understanding of specific details or events, interrupting the patient tactfully when the conversation veers off-topic, gently steering them back to the relevant information with phrases like “Can I bring you back to” or “Can you tell me more about”, utilizing moments of silence effectively, giving the patient space to think and respond without feeling rushed, employing closed-loop communication techniques, explaining concepts or instructions and then asking the patient to repeat them back in their own words to confirm understanding, summarising the information discussed to ensure that the correct events or timeline are understood, and seeking clarification if there are any discrepancies or ambiguities.

Objectively, it is difficult to assume that all doctors possess the communication skills to conduct the medical history interview in accordance with the rules outlined, primarily because in the vast majority of cases they have not received the necessary training. Not to mention the aspect of time, which is of paramount importance: conducting a communicatively effective anamnesis requires time, time that is not available in departments with often hectic activities.

Another limitation of the traditional anamnesis is the fact that it takes place in one go, unlike the digital anamnesis, which involves a “pre-anamnesis” taken using digital instruments (in the waiting room or even at home) followed by the actual anamnesis. The fact that the traditional anamnesis takes place in one go actually makes it more difficult for the patient to recall his or her past medical history effectively. In fact, when it comes to recalling events that took place many years earlier, the patient often needs time to put ideas in order. It is therefore far from uncommon (especially in the case of elderly patients and patients with a very extensive past medical history) for patients to provide incorrect, incomplete, or partial information during the anamnesis.

Another aspect that should not be underestimated is that of the language barrier [13]: very often the taking of a medical history is greatly hindered (sometimes made completely impossible) by the fact that the patient does not speak the language spoken in the country where he or she is being treated. This problem is usually remedied in the traditional anamnesis by having recourse to translators (who may simply be the patient’s family members or duly appointed staff on duty at the hospital) or, in the worst case, by speaking in “universal” languages such as Spanish, the second-most spoken language in the world, or English, ranking third in worldwide prevalence. The language barrier is, however, a fundamental problem of medical history taking, which can lead to miscommunication underlying medical errors [14].

A further weakness of the traditional anamnesis is that the doctor often forgets to ask certain questions, omitting to report some important anamnestic features. Only in some cases, in fact, is the anamnesis collected through the use of pre-filled forms (which act as a “checklist”). In other cases, the doctor simply writes on a blank sheet of paper or at most the titles of the sections to be completed, without specifying the individual aspects to be investigated.

Indeed, it can be difficult for the doctor to remember all the aspects that need to be investigated. Suffice it to say that in order to avoid forgetting, mnemonic techniques have been developed to enable the physician not to forget to investigate important aspects of the anamnesis. The best-known example is the acronym “SOCRATES” for the study of pain characteristics: site, onset, character, radiation, associations, time course, exacerbating/relieving factors, and severity [15]. Other examples concern acronyms that help to remember the individual phases of the history, such as AMPLE (allergies, medications, past medical history, last meal or other intake, and events leading to presentation) and SAMPLE (like AMPLE but with the addition of the S, which stands for signs and symptoms).

Listed below are the aspects that, in the authors’ clinical experience, are most commonly overlooked by the physician taking the traditional history:

- Psychiatric and mental health history: Information on stress, anxiety, depression, mood disorders, and other aspects of mental health;
- Sexual history: Questions related to sexual and reproductive health, safe sex practices, presence of STIs (sexually transmitted infections), etc.;
- In-depth family history: Specific information on inherited diseases, recurring conditions in the family, or causes of death of close relatives;
- Detailed social history: Questions about profession, working environment, substance use (alcohol, tobacco, recreational drugs), living conditions, and social support;
- Complete pharmacological history: Including the use of over-the-counter drugs, food supplements, herbal medicines, and the patient’s adherence to the prescribed regimen.

## 3. The Digital Anamnesis: History, Potential and Critical Issues

A digital anamnesis is a procedure through which the patient answers questions about his or her personal medical history without interacting directly with a doctor, but rather with a technological tool (computer, tablet, applications on mobile phones) through traditional software or chatbots and virtual assistants based on artificial intelligence [16]. 

The first digital anamnesis systems date back to 1949, when a study was conducted at Cornell University that processed paper questionnaires via computer [17]. The Cornell Medical Index, with 195 questions for patients, revealed that this system collected 95 percent more information than the clinical one and was thorough and satisfactory for patients. The use of the computerised questionnaire made it possible to obtain a wide range of data on the patient’s medical history without taking up the physician’s time, facilitating the interview and ensuring that no significant symptoms were overlooked. Of course, at this early stage, the computers were technologically backward, so they were large, located in special rooms, operated with punch cards, and controlled by an information technology specialist. It was not until the 1960s that there was a significant technological advancement in the interface between patient and computer: Slack and others introduced video terminals connected to minicomputers to interview patients about allergies, no longer using punch cards [18]. Since the 1970s, a large number of studies have tested the advantages and disadvantages of computer-generated medical histories. 

Table 1 summarises the characteristics of the most relevant digital anamnesis studies between 1968 and 2001, taken from Bachman’s work [9] (modified and supplemented). 

Today, there are mainly 18 digital instruments [78] used to collect the medical history, the main features of which are summarised in Table 2, listed in chronological order of development. Six of them (Instant medical history, HELP System, AIDA, ParentLink, CIDI-Auto, and MEDoctor) were created between the 1980s and early 2000s, while the remaining 15 were developed more recently, in the last 20 years.

The digitised anamnesis by means of self-compilation by the patient (thus, in fact, a ‘self-anamnesis’, which does not replace but complements the classical anamnesis) offers undoubted advantages for both healthcare professionals and patients.

First of all, patients come to the traditional anamnestic interview better prepared, having already gone through their clinical history and thus having in their memory all relevant information to provide to the doctor. As explained above, in fact, digital anamnestic collection systems do not replace but complement traditional anamnesis, acting in fact as a pre-anamnesis. This facilitates the patient, who has time and a way of recalling his or her medical history, but also the physician, who, having the pre-anamnesis data at his or her disposal, can conduct a more targeted, precise, and detailed anamnesis.

Secondly, the automation of the anamnesis process significantly reduces the administrative burden on healthcare personnel [111], both in terms of time and bureaucracy. This has a significant effect not only on the collection of the anamnesis itself, but also on the entire activity of the physician, who will perform his work with less stress and less risk of burnout [112].

Another aspect of primary importance is that a digital collection of the medical history enables a structured, and thus more complete, recording of the information provided by the patient [113]. It should also be noted that the increasing development of AI (artificial intelligence) systems could bring the efficiency of digital anamnesis to an even higher level in the coming years. AI algorithms can analyse patient responses, identify warning signs, and prioritise critical information for healthcare professionals, thus facilitating efficient decision-making [114].

A final positive aspect to mention is the ease of collecting information that the patient finds embarrassing or ashamed of (e.g., risky sexual behaviour). Interfacing with a digital, non-human instrument undoubtedly facilitates the communication of such aspects pertaining to the private sphere, there being on the other hand no possibility of a moral judgement regarding the patient’s conduct. However, the collection of anamnesis through digital tools also has its downsides.

First, there is the fundamental issue of the so-called “digital literacy” [115,116]. Not all patients are inclined to use digital tools, and not all patients are able to interact effectively with them. It has to be said that the more technology advances, the more effective and engaging the interfaces become, so it is certainly easier today to get patients to adhere to them than it was with the first systems developed in the 1960s.

A second potentially negative aspect of relevance is that whereas in traditional anamnesis the patient is assisted and helped by the physician in reconstructing his or her medical history, in digital anamnesis he or she is in fact alone. This means that there is no guarantee that the patient adequately understands the questions he or she is asked, and there is a risk that he or she may provide inaccurate answers.

Another major negative element is privacy and security [114]. If patient data are collected via digital systems, they are inevitably exposed to possible intrusion by hackers or unauthorised parties. This imposes the need for healthcare facilities to equip themselves with protection systems (antivirus software) that are often very expensive [117].

A further critical aspect related to digital anamnesis is the depersonalisation of the therapeutic relationship. The traditional anamnesis is normally lengthy and time-consuming, but during such an interview an emotional connection is established between the physician and the patient that forms the basis of the therapeutic alliance. The anamnesis collected following the compilation of the pre-anamnesis through a digital tool is certainly quicker and more effective than the traditional anamnesis, but it has the disadvantage of not being long and in-depth enough to allow the establishment of a solid emotional and communicative bond between doctor and patient.

## 4. Bioethical and Medico-Legal Profiles

A first aspect of medico-legal relevance is undoubtedly that of confidentiality. In fact, this aspect is also of bioethical importance, if we consider how sensitive certain types of information can be, and how much the doctor–patient relationship can be affected by a breach of trust in maintaining professional secrecy. In the United States there are specific regulations that protect confidentiality, such as the Health Insurance Portability and Accountability Act (HIPAA), in force since 1996, which enforces the protection and confidentiality of patient data and provides for articulated privacy and security rules (including, for example, encryption and audit logs on access). In Europe, however, there is the EU Regulation 679/2016 (GDPR, General Data Protection Regulation), the privacy regulation that became fully applicable in all EU member states on 25 May 2018. The differences between HIPAA and the GDPR are essentially fourfold [118]: the GDPR provides for greater restrictions on the processing of health data in the absence of patient consent; HIPAA does not provide for the so-called ‘right to be forgotten’, which is covered by the GDPR; HIPAA provides for a much simpler process than the GDPR with regard to the handling of data breaches; the economic penalties are much harsher in Europe (maximum of EUR 20 million or, for companies, 4% of total annual worldwide turnover versus fines of 100,000–250,000 per breach in the US, with an annual cap of USD 1.5 million). What is crucial to do when using tools such as those that make digital anamnesis possible is to invest significantly in IT security systems and staff training. This means implementing robust security measures such as firewalls, antivirus software, and intrusion detection systems, as well as ensuring that mobile devices used to transmit confidential information are properly encrypted.

A second aspect of purely bioethical interest is that relating to the characteristics of the doctor–patient relationship, the physiognomy of which is evidently modified by the use of digital tools for the collection of the anamnesis, which represents the first act in the relationship between the two protagonists of the therapeutic alliance. Although originally developed in the field of psychotherapy [119], the therapeutic alliance has gradually asserted itself, together with the overcoming of the paternalistic model, as the ideal model of empathic relationship between doctor and patient, and since the collection of the clinical history represents the first moment of contact between these two figures, it is legitimate to wonder whether depersonalising it might not run the risk of jeopardising the establishment of an effective relationship of mutual trust between the healer and the treated. This is indeed a real problem, considering that a physician’s knowledge of the data emerging from the outcome of the digital ‘pre-anamnesis’ will undoubtedly lead them to shorten the interview time, and above all to conduct a ‘surgical’, targeted, selective anamnestic examination. This accelerated mode of collecting the medical history undoubtedly entails a major saving in terms of time and effort, as well as obvious advantages in organisational terms (more time and energy to devote to the care of other patients), but it puts the aspect of empathic communication in the background, if only for mere temporal reasons (establishing an empathic relationship takes time). It is therefore necessary to ask oneself whether, from an overall perspective, one can consider oneself willing to accept a reduction in the probability of establishing an effective therapeutic alliance in order to benefit from time and energy savings and enjoy an optimisation of the organisation of healthcare. It should also be considered how the flattening of the doctor–patient relationship can be particularly important in certain socio-cultural fabrics, such as rural communities, where doctors are often not only health professionals but also integral members of the local community. Here, the doctor–patient relationship transcends the purely clinical realm, intertwining with community dynamics and cultural expectations. The erosion of personalised interaction facilitated by digital tools may not only hinder the establishment of trust and rapport, but also weaken the community fabric in which the physician plays a central role. The loss of this interpersonal bond could be perceived as a decrease in community cohesion and could lead to a sense of alienation among patients who rely not only on medical expertise, but also on the emotional support and solidarity provided by their local healer. It is therefore imperative to recognise that the implications of communication flattening vary in different healthcare contexts and underline the need for nuanced and context-sensitive considerations when implementing digital solutions in medical practice.

A third and final aspect that is important to highlight as it has important implications on both the bioethical and medico-legal fronts is that of safety of care. Digital medical history collection can only be used effectively and safely if it can guarantee the acquisition of real and reliable data. In contrast to a clinical interview conducted by a physician, a computerised anamnestic collection poses problems relating on the one hand to the patient’s digital literacy (his familiarity with the use of technological tools) and on the other hand to his health literacy (his degree of knowledge of medical terminology and medicine in general). The first problem is probably relatively easy to solve, since, as shown by the above-mentioned literature, the development of increasingly simple, attractive, and engaging interfaces makes it possible in the vast majority of cases to overcome the obstacles related to the unfamiliarity of users (especially older users) with technology. The second problem, on the contrary, appears in our opinion to be more complex to solve. How can one be sure that the patient adequately understands the meaning of the questions posed to him by a digital system with which he cannot interface as directly as he would with a human? How can the digital system confirm that the patient’s answer is not the result of a blatant misinterpretation? How can a computerised system adapt the type of language and the way medical information is explained to the degree of culture and preparation of the individual patient? These are questions that currently remain with a question mark. Indeed, the paradoxical scenario cannot be ruled out whereby a physician faced with a patient with a limited technological proficiency background does not trust the information resulting from the digital pre-anamnesis at all and prefers to take a traditional anamnesis from scratch, with the absurd result that instead of saving time, time would be lost.

Again, with regard to safety of care, it should not be overlooked that there is a risk that a systematic use of digitised medical history collection systems could lead doctors to over-rely on digital technologies, which could be accompanied by a decrease in clinical skills. If physicians rely too much on results generated by algorithms and chatbots, they might neglect to carefully examine patients and consider other important factors in the diagnosis and treatment of diseases that are not already considered by digital systems. In other words, a kind of ‘laxity’ of doctors could occur, which could lead to a decrease in the quality of care provided and an increase in malpractice cases.

The issue of safety of care is intimately connected with the issue of medical liability. Should malpractice occur due to errors in anamnestic collection, who would be held liable: the doctor, the software developer, the software programmer, or the company marketing the device? This is a thorny issue that has been widely debated for several years. The European Parliament and the Council are in the process of finally adopting the proposal for a regulation presented by the European Commission on 21 April 2021, on a legal framework for artificial intelligence (‘Artificial Intelligence Act’). Both the product liability directive and the artificial intelligence non-contractual liability directive are under discussion. Not all digital anamnestic collection systems are based on artificial intelligence, but the liability profiles applicable to such systems may in many ways appear overlapping, since they are non-human entities interacting with patients and actively involved in the diagnosis and treatment process.

The issue of liability from digitised collection of informed consent would actually be much easier to handle than liability from malfunctioning of devices physically operating on the patient (such as robots used in surgery). This is because the activity provided by digital anamnestic collection systems is purely intellectual, which evidently implies that it is much more easily controlled and governed by human doctors. It is quite clear that a human surgeon has very little (if any) control when faced, for example, with a mechanical arm of a robot that autonomously performs (on the basis of AI algorithms) an incorrect manoeuvre causing injury to an anatomical structure. It is equally evident how an omitted or erroneous anamnestic information represents, on the contrary, an error that is much easier to remedy, also considering the fact that digital anamnesis, as widely observed, represents only a complement to the traditional anamnesis, not replacing it. It therefore seems legitimate to argue that the greater the possibility on the part of the physician to control and govern the work of the non-human system, the greater the liability profiles for the physician. In the case of the digital anamnesis, therefore, it is plausible to assume that the heaviest burden of responsibility would be on the physician, who in any case has the burden of verifying that the information assumed by the digital collection system is correct and true, and above all that no important information has been overlooked. However, it should be pointed out that if the physician were to go through the traditional anamnesis again to verify the reliability of every single piece of information collected by the digital system, the digital anamnesis would lose its meaning, leading to no advantage in terms of saving time and work for the physician (who would basically be forced to take a traditional anamnesis from the beginning in order to avoid any liability charges).

This is an issue that is difficult to resolve, since the fact that the final responsibility for the adequacy of the anamnestic collection falls on the physician makes the latter obviously interested in ensuring that the preliminary anamnestic collection made by the digital system is correct and accurate, and this would precisely lead to the nullification of the advantage of time and effort that the use of the non-human system would bring, since the physician would take a new anamnesis from scratch (with the risk, moreover, of collecting less reliable information than that collected by the digital system in the first instance, since the patient would be called upon to repeat his or her entire medical history from the beginning, and would therefore be more fatigued and less attentive, and thus more prone to making mistakes).

## 5. Conclusions

In conclusion, in the authors’ opinion, it is completely unreasonable to halt technological progress by hindering the development and dissemination of technological tools that can concretely facilitate the work of healthcare professionals, saving them time and energy. What needs to be done, rather, is to attempt to redefine the established structures of the doctor–patient relationship in such a way as to make it adhere to the new clinical reality.

As has emerged from the outcome of the literature review, the most concrete issues related to the implementation of digital anamnesis systems are those of communication flattening and of safety of care in relation to the treatment of digitally illiterate patients. These challenges have to be addressed proactively, e.g., by fostering the development of new communication skills on the part of physicians and by developing digital systems that are able to compensate more and more effectively for the gaps (digital or medical knowledge) of patients.

## Figures and Tables

**Table 1:** The main digital anamnesis tools developed from the 1970s to the 2000s.

Authors and Year	Brief Description	Number of Patients	Time Required for Anamnesis	Human or Computer Better?
Mayne et al., 1968 [19]	System for interviewing by means of an expensive state-of-the-art computer, using an optical pen	159	66 min	Computer
Coombs et al., 1970 [20]	Study of the factors influencing patients’ performance in automating their medical histories	145	24.1 min	/
Grossman et al., 1971 [21]	Evaluation of the effectiveness of computer-generated medical histories	500	/	Computer
Stead et al., 1972 [22]	Using computer assistance to interview patients with functional headache	50	/	Computer
Greist et al., 1973 [23]	Development of a computerised interview to predict suicide risk	22	90 min	Computer
Pearlman et al., 1973 [24]	Creation of an automated questionnaire for monitoring the health of newborns	71	17.8 min	/
Evans and Gormican, 1973 [25]	Design of a system to collect data on the diet of diabetics	50	63 min	Human
Card et al., 1974 [26]	Comparison of patients’ questions between doctor and computer regarding dyspeptic symptoms	72	/	/
Bailey [27]	Questioning the usefulness of pre-employment medical examinations through the use of a computerised health questionnaire	/	/	/
Schuman et al., 1975 [28]	Implementing a computerised life event interview	93	15–20 min	/
Lucas et al., 1976 [29]	Comparison of patients’ questions between doctor and computer regarding dyspeptic symptoms	75	/	Computer
Chun et al., 1976 [30]	Using computerised interviews for patients with epilepsy	32	80 min	Computer
Angle et al., 1977 [31]	Introduction of computer-assisted interviewing in behavioural analysis	331	240–480 min	/
Lucas et al., 1977 [32]	Comparing the effectiveness of psychiatrists and computers in interviewing patients with alcohol-related illnesses	36	26 min	Computer
Hastings and Whitcher, 1979 [33]	Development of an automated medical screening in an urban prison	20	/	/
Tompkins et al., 1980 [34]	Evaluation of the usefulness of a computer-assisted pre-anaesthesia interview	84	45 min	Computer
Bana et al., 1980 [35]	Development of a computer-assisted interview for headaches	40	/	/
Rudicel and Jokl, 1981 [36]	Application of a computer-generated pre-participation medical history collection system for athletes	20	<30 min	/
Carr et al., 1981 [37]	Study of the direct assessment of depression using microcomputers	168	/	/
Lilford and Chard, 1981 [38]	Study of the use of microcomputers in prenatal care, analysing the feasibility of the initial interview	/	11–13 min	/
Carr et al., 1983 [39]	Investigation of whether a computer could collect a psychiatric history	37	/	Computer
Skinner and Allen, 1983 [40]	Comparation of computer-based assessment with face-to-face and self-assessment for alcohol, drug, and tobacco use	150	/	Human
Millstein and Irwin, 1983 [41]	Evaluation of the acceptability of computer-acquired sexual histories in adolescent girls, investigating the impact of technology in the area of sexual health	108	/	Computer
Lilford et al., 1983 [42]	Use of an interactive microcomputer system to collect clinical histories in a gynaecological endocrinology and infertility clinic	200	27 min	/
Trell, 1983 [43]	Introduction of an interactive programme for the distribution of medical questionnaires	10,000	15–30 min	/
Leviton et al., 1984 [44]	Development of a computerised behavioural assessment for children with headaches	69	/	/
Bingham et al., 1984 [45]	Exploration of the strengths and weaknesses of direct patient interviewing using a microcomputer system in a specialist gynaecological practice	190	21–27 min	Computer
Quaak et al., 1986 [46]	Comparison of computerised and traditional medical records	99	60 min	Computer
Farrell et al., 1987 [47]	Identification of target psychic disorders by means of a computerised interview	103	30 min	/
Glen et al., 1989 [48]	Examination of urological history and management recommendations via microcomputer	262	11–13 min	/
Bernadt et al., 1989 [49]	Assessing the reliability of a computer in detecting an alcohol history	102	/	No difference
Levine et al., 1989 [50]	Examining suicide risk assessment by means of self-administered computer questionnaires	102	/	Computer
Paperny et al., 1990 [51]	Identification of and intervention in adolescent health risk behaviour using computers	3327	/	Computer
Adang et al., 1991 [52]	Evaluation of computerised questionnaires for patients awaiting gastrointestinal endoscopy	362	11 min	/
Lutner et al., 1991 [53]	Comparison of automated interviews with in-person interviews, analysing differences in patients’ answers to questions on preoperative health	/	239 min	/
Lapham et al., 1991 [54]	Computer-based screening for behavioural risks in pregnancy in an antenatal clinic	265	/	/
Locke et al., 1992 [55]	Using a computer-based interview to screen blood donors for risk of HIV transmission	294	8 min	Computer
Roizen et al., 1992 [56]	Exploring the ability of patients to use an automated questionnaire to define their health status, improving health self-management	250–262	/	/
Robinson and West, 1992 [57]	Comparing computerised methods and questionnaires for the collection of medical history in a genito-urinary clinic	49	/	Computer
Wenner et al., 1994 [58]	Presentation of “Instant Medical History”, a knowledge-based patient driven screening expert system which simplified the collection of medical histories	10,000	/	/
Petrie and Abell [59]	Studying the responses of individuals who attempted suicide to a computerised interview	150	/	Computer
Boekeloo et al., 1994 [60]	Comparing audio and written questionnaires for the reporting of HIV risk factors by patients in a sexually transmitted disease clinic	305	6 min	Computer
Slack et al., 1995 [61]	Implementing a computer-administered health screening interview for hospital staff	1987	80 min	/
Hasley, 1995 [62]	Comparing computerised and personal interviews for updating gynaecological history	200	/	Computer
Wald et al., 1995 [63]	Using an interactive interview in the electronic medical record to collect data from patients in primary care	172	27	/
C’De Baca et al., 1997 [64]	Using computerised interviews to test associations between risk factors and pregnancy outcomes	197	/	Computer
Kohlmeier et al., 1997 [65]	Introduction of computer-assisted self-interviewing as a multimedia method for dietary assessment	/	/	/
Kobak et al., 1997 [66]	Performing computer-aided screening for psychiatric disorders in a community mental health clinic	51	/	Computer
Newell et al., 1997 [67]	Evaluating the acceptability of computerised touch-screen surveys among medical oncology patients	229	15 min	/
Kim et al., 1997 [68]	Empirical study on the Health Status Questionnaire System for patient-computer interaction	112	/	/
Hunt et al., 1997 [69]	Study of the applicability of automation systems in the collection of information directly from patients with diabetes	47	15	Human
McRoy et al., 1998 [70]	Exploring computer-based interactive health education	/	/	/
Buxton et al., 1998 [71]	Documenting patients’ experiences in using a computerised programme for health-related quality of life assessment	178	7 min	/
Shakeshaft et al., 1998 [72]	Investigating the acceptability of computers in community-based clinical settings for addictions	179	/	/
Williams et al., 1998 [73]	Testing a patient initiative system for preventive health promotion	557	/	/
Kissinger et al., 1999 [74]	Application of computer-assisted interviews to research on sexual behaviour	280	/	Computer
Reilly, 1999 [75]	Examining the symptom experience of hospitalised patients using a pen computer	72	/	/
Pierce, 2000 [76]	Studying the use of “Instant Medical History”, a knowledge-based patient driven screening expert system, in a rural clinic	25	/	/
Rhodes et al., 2001 [77]	Controlled study on a computer-based intervention for health screening and promotion in the emergency department	248	15–18	Computer

**Table 2:** The main digital anamnesis tools used in clinical practice today.

Tool	Key Characteristics
Instant medical history [58,78,79]	– Developed by Primetime Medical Software Inc in 1985 – Designed to obtain comprehensive information about the history of present illness, saving physician time and making documentation more complete – Patients select a chief complaint through a web-based portal and answer multiple-choice questions about their symptoms – Information is submitted to the electronic health record for review before the patient visit – Reported to save up to 6 min per clinical encounter – Used in 7 countries by 44,500 physicians, estimated to be used in 80 million visits in 2020
HELP System [78,80,81]	– Programmed on the Microsoft Query driver, described in a 1987 publication – Integrated into the hospital’s HELP information system, pioneering clinical decision support – The tool’s list of 5 differential diagnoses included the principal discharge diagnosis for 85% of the patients in a study – Formed the basis for a subsequent diagnostic application, Iliad, used in medical education – No longer in use within the hospital setting
AIDA [78,82,83]	– Developed in 1987 by the department of medical informatics at Erasmus University, Rotterdam – Aimed to automate medical history taking to aid physicians in arriving at an accurate diagnosis – Patients read questions on a screen and press keys corresponding to their answers, with the system containing over 400 questions relating to 179 different items – Favourably rated by users in terms of usefulness – Was not further developed into a product used in routine care
ParentLink [78,84,85]	– Developed in 1989 – Aimed at collecting children’s symptoms information from parents in the paediatric emergency department – Allows parents to submit data through an electronic terminal, covering structured question pathways or open-ended free text – Parental entry averages 5 min, with data quality comparable to physicians’ documentation – Improved sensitivity in parental documentation for hydration status noted – Has been utilized in studies for various paediatric complaints beyond its initial scope
CIDI-Auto [78,86,87,88]	– Computerized version of WHO’s Composite International Diagnostic Interview for psychiatric differential diagnoses – Developed in 1997 – Patients answer yes-or-no questions on psychiatric symptoms at a computer workstation, taking approximately 75 min – Psychiatric physicians found 50% of the CIDI-Auto current diagnoses agreeable – Reported as a potential aid for indirect or remote diagnosis – Has evolved into the WHO World Mental Health-CIDI Instrument, administered by computer globally
MEDoctor [78,89]	– Developed in 1999 – A symptom checker tool designed to generate a list of differential diagnoses based on user input – Utilizes a rule-out method and Bayesian statistics to navigate through >4200 symptoms – Provides users with a list of top 3 differential diagnoses and a detailed report of responses – Diagnostic agreement with vignettes is measured – Used worldwide for over 5 years, with thousands of completions across multiple countries
CLEOS [90,91]	– Introduced in 2008 to enhance patient history taking and support clinical decision-making – Utilizes 450 decision trees representing medical knowledge to explore significant history aspects – In a 2008 study, detected 3.5 additional problems per patient on average – Currently tested in a clinical trial at Karolinska University Hospital for evaluating patients with chest pain – Used at Danderyd University Hospital in Stockholm, Sweden, in a clinical trial context
Mediktor [78,92]	– Launched in 2011 as a symptom-checker leveraging AI and natural language processing – Offers conversational-style prompts or multiple-choice questions, assessing triage urgency and possible diagnoses – Diagnostic accuracy matched physician diagnosis in 91% of cases in a Spanish study – Available through Amazon’s Alexa and Telegram, with 1.2 million evaluations in 2019 – Used at 3 clinical sites in Europe and the United States
DocResponse [78,93]	– A patient intake and documentation tool developed in 2012 to streamline clinical workflows – Allows patients to enter data on smart devices, providing clinical decision support to clinicians – Found to be the symptom checker most likely to arrive at the correct principal diagnosis in a 2015 study – Used at >170 clinical sites across various specialties in the United States – Supported >225,000 encounters in 2019, according to the vendor
Digivey [78,94]	– Developed in 2013 – Self-administered interview tool designed to improve diagnostic accuracy and patient safety – Utilizes adaptive questionnaires on mobile kiosk, touch-screen monitor, or laptop – Deemed usable with low rates of user error, taking about 6 min to complete – Used in research studies, including a 5-site multicentre clinical trial screening > 3000 patient encounters – Johns Hopkins department of neurology plans to deploy it in several clinical areas
PatientTouch [78,95]	– Developed in 2014 by PatientSafe Solutions Inc for symptom description via electronic questionnaire – Used handheld touch-screen tablet with chief complaint-specific algorithms in English or Spanish – Patients reported positive experiences, believing the device would improve communication and care quality – Not currently used at any clinical sites
OurNotes [78,96,97]	– Developed in 2015 – Part of the OpenNotes movement, allowing patients to contribute to ambulatory visit notes – Patients submit answers to open-ended questions before visits, which are incorporated into visit notes – Pilot study showed patient support and potential clinician workload reduction – Pilot involved 160 primary care clinicians and 2500 patients across 4 academic medical centres between 2018 and 2020. – Evaluation and potential expansions ongoing
FirstHx [78,98,99]	– Patient intake tool developed in 2016 for use in emergency, urgent care, and telemedicine settings – Utilizes a physician-like questioning process covering > 240 complaints in up to 10 languages – Digital intake process takes 3–6 min on smartphones, tablets, or kiosks – Available in the Epic App Store, pilot tested at >10 sites – Estimated to support 600,000 visits per year
Automated Evaluation of Gastrointestinal Symptoms (AEGIS) [78,100,101]	– Developed in 2016 to automate gastrointestinal symptom reporting in clinics – Web portal facilitates patient symptom characterization based on the PROMIS framework – Generates comprehensive physician-facing reports mirroring traditional histories – Proven to produce more complete and useful documentation than usual care – Superior in detecting alarm features compared to physician detection – Not used in routine patient care beyond studies
DCAT [78,102,103,104]	– Anamnesis tool created in 2017, designed to improve communication in primary care, especially for refugee care in Germany – Tablet-based symptom and medical history data entry, with visual and audio aids for low literacy users – Translates patient data into clinician’s preferred language, highlighting red flags – Demonstrated good usability and acceptance across multiple languages – Used in 10,000 multilingual visits in 2021
Quro [78,105]	– A chatbot health assistant launched in 2017 by Medius Health, utilizing AI for health assessments – Engages users through free-response and multiple-choice questions, leveraging a large clinical knowledge graph – Provides users with a list of differential diagnoses, urgency recommendations, and detailed reports – Triage accuracy assessed as 83% in case-based scenarios – Marketed to health service providers for remote patient engagement
Mandy [78,106]	– Developed in 2017 by a research partnership in Auckland, New Zealand, for primary care – Utilizes conversational prompts and natural language processing for patient intake – Analyses patient responses to generate further interview questions and a differential diagnosis – Demonstrated ability to generate appropriate questions and predict diagnoses with varying accuracy
Ana [16,107]	– Developed in 2018 – Introduces a concept of a self-anamnesis implemented as a mobile application for patients – Uses a conversational user interface to simulate the conversation between patient and therapist, improving the interaction and accuracy of the information collected – Equipped with 63 questions posed successively to the user, using a rule-based approach and Artificial Intelligence Markup Language (AIML) to manage the chatbot conversation – Offers advantages over traditional digital questionnaires, such as encouragement to complete all questions and the possibility for the user to ask for clarification – Integrated into the care process, allowing collected data to be stored in a structured manner on an eHealth platform, making it accessible to therapists or physicians for a better information base for initial consultation and subsequent decision-making
Diagnosis and Anamnesis Automated Medical History–Taking Device (DIAANA AMHTD) [78,108]	– Developed in 2019 – Aimed at improving diagnostic accuracy for musculoskeletal complaints, developed by Logic-Based Medicine Sàrl and Lausanne University Hospital – Piloted in Geneva, Switzerland, involving a touch pad-based adaptive questionnaire for patients – Generated comprehensive anamnesis summaries and differential diagnoses for physician consideration – Showed improved diagnostic inclusion by residents – In use by physicians and the Swiss telemedicine system
Digital Structured Self-Anamnesis Tool for CT patients [109]	– Developed in 2020 to evaluate the performance of a structured digitised self-assessment (DSSA) of the patient’s medical history (PA) prior to a computed tomography (CT) examination – Consists of a tablet-based questionnaire of 67 items covering social history, lifestyle factors such as tobacco abuse, medical history such as kidney disease, current symptoms, and system usability – Allows patients to mark unclear questions for later discussion with the radiologist and automatically highlights critical problems for CT examination as ‘red flags’ – Feedback from patients regarding the comprehensibility of the questionnaire and the usability of the tablet was predominantly positive (90.9%; 86.2%), with a completion time of less than 20 min for 85.1% of patients
App for digital medical history taking in urgent care practices [110]	– Designed to collect patients’ medical history based on symptoms in out-of-hours urgent care settings – Allows patients to select their acute symptoms and guides them through a series of related questions, such as specific symptoms, relevant pre-existing conditions, and previous treatments or medications – Questions and answers are formulated in plain language, with a simple design and intuitive navigation, making the app easy for patients to use without prior instruction. After completion, it generates a structured medical history summary that can be transferred to the patient’s electronic medical record (EMR)

## Data Availability

The original contributions presented in the study are included in the article material; further inquiries can be directed to the corresponding author.
